# Microwave Specular Measurements and Ocean Surface Wave Properties

**DOI:** 10.3390/s21041486

**Published:** 2021-02-20

**Authors:** Paul A. Hwang, Thomas L. Ainsworth, Jeffrey D. Ouellette

**Affiliations:** Remote Sensing Division, U.S. Naval Research Laboratory, Washington, DC 20375, USA; ainsworth@nrl.navy.mil (T.L.A.); jeffrey.ouellette@nrl.navy.mil (J.D.O.)

**Keywords:** ocean surface roughness, normalized radar cross section, specular reflection, relative permittivity, whitecaps

## Abstract

Microwave reflectometers provide spectrally integrated information of ocean surface waves several times longer than the incident electromagnetic (EM) wavelengths. For high wind condition, it is necessary to consider the modification of relative permittivity by air in foam and whitecaps produced by wave breaking. This paper describes the application of these considerations to microwave specular returns from the ocean surface. Measurements from Ku and Ka band altimeters and L band reflectometers are used for illustration. The modeling yields a straightforward integration of a closed-form expression connecting the observed specular normalized radar cross section (NRCS) to the surface wave statistical and geometric properties. It remains a challenge to acquire sufficient number of high-wind collocated and simultaneous reference measurements for algorithm development or validation and verification effort. Solutions from accurate forward computation can supplement the sparse high wind databases. Modeled specular NRCSs are provided for L, C, X, Ku, and Ka bands with wind speeds up to 99 m/s.

## 1. Introduction

The range of ocean surface wavelengths important to microwave remote sensing extends several orders of magnitude. Crombie [1] reports the Doppler frequency spectrum of 13.56 MHz radar sea echo at low grazing angle. A distinct spectral peak at 0.38 Hz is illustrated, corresponding to the resonance ocean surface wavelength of about 10 m (wavenumber *k* about 0.6 rad/m). He goes on to suggest that variable frequency equipment can be used to measure the ocean surface wave spectrum. Depending on frequency and incidence angle, the range of resonance surface wavenumbers spans from about 20 rad/m (L band) to about 500 rad/m (Ku band) for microwave sensors operating at moderate and high incidence angles [2,3].

For altimeters and reflectometers, the specular reflection mechanism dominates. The NRCS is proportional to the number of specular points and the average radii of curvature of the specular reflection facets [4,5,6]. With the Gaussian distribution describing the elevation and slope of the moving ocean surface [7], a simple inverse relationship between NRCS and surface mean square slope (MSS) is established [5,6]. Further analysis [8,9,10] indicates that the responsible MSS is contributed by surface waves longer than the EM wavelengths. The frequently cited ratio between the EM wavenumber *k_r_* and the upper cutoff wavenumber *k_u_* of Low-Pass MSS (LPMSS) integration is between 3 and 6 [9,10]. Thus, for Ku band (~14 GHz) altimeter, *k_u_* is about 50 to 100 rad/m, and for L band (~1.6 GHz) reflectometer it is about 6 to 11 rad/m.

These theoretical and empirical analyses provide useful guidelines for quantitative investigation of the connection between specular NRCS and ocean surface roughness. Ku band altimeters have been in operation for many decades, and there is a rich trove of well-calibrated Ku band altimeter NRCS data for a close examination of the specular point theory (SPT) applied to nadir-looking altimeters: $\sigma_{0}={\left|R\left(0\right)\right|}^{2}/s_{f}^{2}$, where *σ*_0_ is NRCS, *R*(0) is the Fresnel reflection coefficient for normal incidence, and $s_{f}^{2}$ is the Ku band LPMSS [8,9,10]. One peculiar outcome is that the resulting $s_{f}^{2}$ calculated from measured Ku band NRCS is larger than the optical total MSS [11,12]. The difference is especially obvious in low and moderate wind speeds (*U*_10_ less than about 10 m/s). To address this paradox, an effective reflectivity ranging from 0.34 to 0.5 has been suggested [8,9,10]; those numbers are much smaller than the nominal relative permittivity of 0.62 computed for the Ku band frequency. An alternative explanation is that the peculiar result can be reconciled if the tilting effect of the reflecting specular facets is considered when applying the SPT [13,14]. It has been about two decades since the study presented in [13,14] and our understanding of the ocean surface wave spectrum has advanced considerably with incorporation of remote sensing data into the relatively sparse databases of short-scale ocean surface waves accumulated from direct observations [15,16,17]. Here we revisit the Ku band altimeter NRCS analyses. The results are applied to other frequencies including the observations of L band LPMSS [18,19,20,21] and recent reports of NRCS results derived from the CYclone Global Navigation Satellite System (CYGNSS) mission [22,23,24], and Ka band altimeter NRCS from the Satellite ARgos and ALtiKa (SARAL) mission [25,26]. The overall objectives are (a) to use specular microwave returns to understand the mean square slopes of surface waves several times longer than the radar wavelengths; spaceborne altimeter and reflectometer measurements coupled with the SPT are employed for this study; and (b) to supplement the high wind in situ data with accurate forward computation of the altimeter and reflectometer solutions.

Section 2 gives a brief review of the SPT [4,5,6,8]. Section 3 describes its application to spaceborne microwave observations. Section 4 discusses issues such as wind speed and wave age relationship on LPMSS, whitecap effects caused by wave breaking on surface reflectivity in high winds, and extending the analysis to various frequencies. The NRCS dependence on incidence and scattering angles are presented and discussed with computed examples; the limitations and range of application are described. Section 5 is summary.

## 2. Review of Specular Point Theory

Kodis [4] presents a theoretical analysis of backscattering from a perfectly conducting 1D irregular surface at very short EM wavelengths (Kirchoff approximation), with the application of the stationary phase principle to the Kirchoff integral for the complex scattered field. The integral formulas are derived directly from the vector field theory. He shows that to the first order approximation, the backscattering cross section is proportional to the product of the average number of specular points illuminated by the EM waves *n_A_*, and the geometric mean of the two principal radii of curvature of those specular points *r*_1_ and *r*_2_, i.e.,

(1) $$\sigma \left(k_{i},-k_{i}\right)∼\pi \langle \left|r_{1}r_{2}\right|\rangle n_{A}$$

where *k_i_* is the incidence EM wavenumber. This analysis elicits the close connection between EM scattering and statistical and geometrical properties of the rough surface. In order to carry out the calculation further, it is necessary to specify the statistics of the rough surface, in particular in regard to the average number of illuminated specular points and their average curvature.

**Figure 1:**
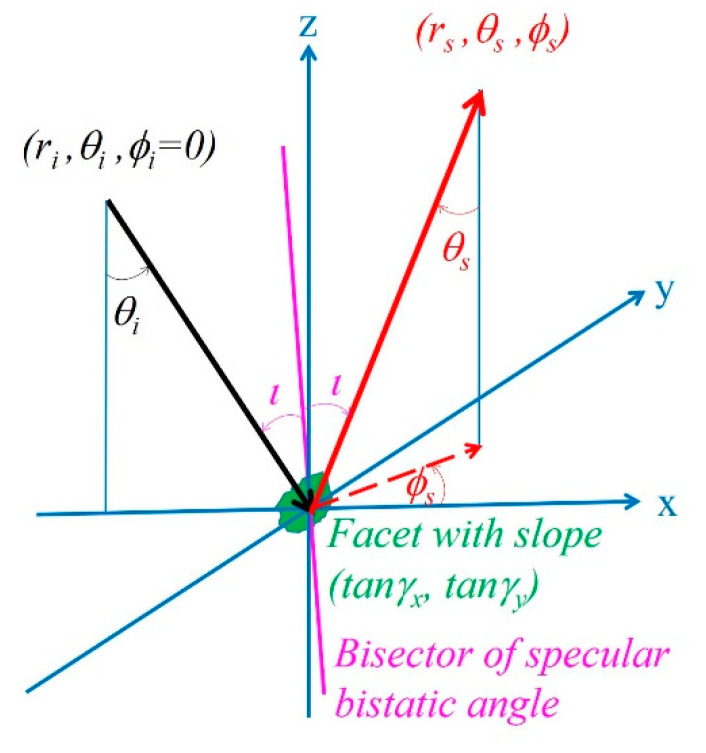
The scattering geometry from a surface facet.

Barrick [5,6] extends the analysis to 2D horizontal plane, full scattering geometry configuration, polarization states, and finite surface conductivity. Following his notations as defined by the scattering geometry depicted in his Figure 1, which is simplified and reproduced here as Figure 1, the NRCS for arbitrary incident and scattered polarization states (*η* and *ξ*, respectively), incidence angles (*θ_i_*, *ϕ_i_* = 0), and scattering angles (*θ_s_*, *ϕ_s_*) is

(2) $$\sigma_{0\xi \eta }^{}=\pi {\left|R_{\xi \eta }\left(\iota \right)\right|}^{2}\langle \left|r_{1}r_{2}\right|\rangle n_{A}$$

where *R_ξη_*(*ι*) is the reflection coefficient from infinite plane tangent to the surface at the specular points for incidence and scattered states, and *ι* is the local (effective) incidence angle at the specular points. From Kodis’s stationary phase analysis [4], it is shown that the effective incidence angle *ι* is half the angle between the incidence and scattering propagation directions [5], and it can be expressed as a function of incidence and scattering angles (after correcting a couple of typographic errors):

(3) $$\cos\iota ={\left[\left(1-\sin\theta_{i}\sin\theta_{s}\cos\phi_{s}+\cos\theta_{i}\cos\theta_{s}\right)/2\right]}^{1/2}$$

The average number of specular points per unit area for a rough surface and the average radii of curvatures are then derived in terms of the surface statistics. Section 2 and Section 3 of Barrick [5] and Appendix B of Barrick [6] give the detailed mathematical derivation of *n_A_* and <|*r*_1_*r*_2_|>. The final results are copied here:

(4) $$n_{A}=\frac{7.255}{\pi^{2}l^{2}}\exp\left(\frac{-\tan^{2}\gamma }{s_{f}^{2}}\right)$$

(5) $$\langle \left|r_{1}r_{2}\right|\rangle =\frac{0.138\pi l^{2}}{s_{f}^{2}}\sec^{4}\gamma$$

where *l* is the correlation length between surface points *ζ*(*x*, *y*) and *ζ*(*x*′, *y*′) separated by a horizontal distance *r* = [(*x*−*x*′)^2^ + (*y*−*y*′)^2^]^1/2^ and assuming a Gaussian distribution of the correlation coefficient as *r* → 0. The numerical constants in Equations (4) and (5) result from carrying out triple integral functions of (*ζ_xx_*, *ζ_xy_*, *ζ_yy_*); the details are provided in Barrick [5,6].

Substituting Equations (4) and (5) into Equation (2), then

(6) $$\sigma_{0\xi \eta }^{}={\left|R_{\xi \eta }\left(\iota \right)\right|}^{2}\frac{\sec^{4}\gamma }{s_{f}^{2}}\exp\left(\frac{-\tan^{2}\gamma }{s_{f}^{2}}\right)$$

where $s_{f}^{2}$ is the ocean surface LPMSS, and tan*γ* is the surface slope at the specular point, which can be expressed as a function of incidence and scattering angles.

(7) $$\tan\gamma =\frac{{\left(\sin^{2}\theta_{i}-2\sin\theta_{i}\sin\theta_{s}\cos\phi_{s}+\sin^{2}\theta_{s}\right)}^{1/2}}{\cos\theta_{i}+\cos\theta_{s}}$$

A case of special interest is backscattering: *ϕ_s_* = π, *θ_s_* = *θ_i_*, *γ* = *θ_i_*, and the NRCS is

(8) $$\sigma_{0\xi \eta }={\left|R_{\xi \eta }\left(\iota \right)\right|}^{2}\frac{\sec^{4}\theta_{i}}{s_{f}^{2}}\exp\left(\frac{-\tan^{2}\theta_{i}}{s_{f}^{2}}\right)$$

## 3. Application

### 3.1. Ku Band Altimeter Analysis

For a nadir-looking altimeter (*θ_i_* = *θ_s_* = *ι* = 0) with linear polarization (*h* or *v* for horizontal or vertical), Equation (8) becomes

(9) $$\sigma_{0}=\frac{{\left|R\left(0\right)\right|}^{2}}{s_{f}^{2}}=\sigma_{0hh}=\sigma_{0vv}$$

where *R*(0), shorthand for *R_ηη_*(0) with *η* = *h* or *v*, is the Fresnel reflection coefficient for normal incidence, and the NRCS is independent on the polarization state. Applying Equation (9) to Ku band altimeter measurements, a rather peculiar result is discovered [8,9,10,13,14]: the computed Ku band LPMSS is larger than the total optical MSS in clean water $s_{\infty }^{2}$ [11,12].

The LPMSS $s_{f}^{2}$ is an integrated surface wave property, which is defined as

(10) $$s_{f}^{2}=\int_{0}^{k_{u}}k^{2}S\left(k\right)dk$$

where *S* is the surface wave elevation spectrum, *k* is surface wavenumber, and *k_u_* is the upper limit of lowpass filter, which is in turn proportional to the EM wavenumber *k_r_*. The ratio *k_r_*/*k_u_* is generally given as between 3 and 6 [9,10]. When distinction of EM frequency is desired, $s_{f}^{2}$ is also given as $s_{ku}^{2}$ in this paper. For example, for Ku band EM frequency *f_r_* = 14 GHz and *k_u_* = *k_r_*/3 = 293/3 = 98 rad/m, the corresponding $s_{f}^{2}$ is $s_{98}^{2}$ for clarification. The optical EM frequency is many orders of magnitude higher than those of the microwave sensors used in ocean remote sensing, so $s_{\infty }^{2}$ is expected to be the upper bound of $s_{f}^{2}$ observed by microwave equipment.

**Figure 2:**
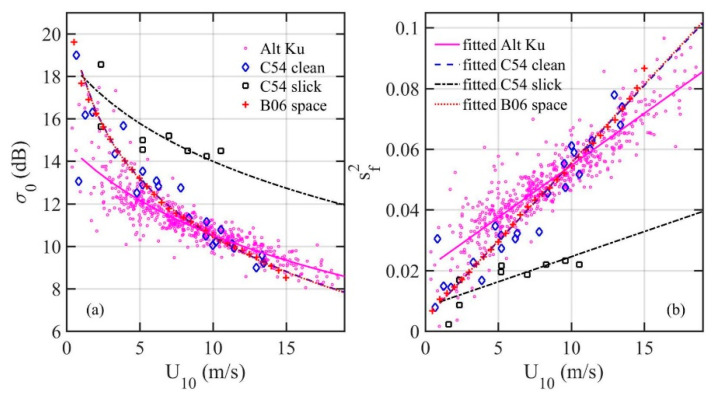
(**a**) Ku band altimeter NRCS and comparison with computational results using Equation (9) with the optical MSS in clean and slick waters: C54 and B06. (**b**) The MSS computed using Equation (9) with the Ku band altimeter NRCS and comparison with the optical MSS in clean and slick waters. The smooth curves are linear fitting to the three sets of MSS data. The smooth curves in (**a**) are the NRCS computation using Equation (9) with the linear fitted curves of the MSS data.

Figure 2a shows the Ku band altimeter NRCS with collocated buoy winds in the Gulf of Alaska and Bering Sea [14], and the calculated NRCSs based on the optical MSS from sun glitter analyses in clean and slick waters with wind speed measured onboard a research vessel in the region [11] or collocated spaceborne scatterometer wind product [12]. Figure 2b shows the optical MSS and $s_{f}^{2}$ derived from the Ku band altimeter NRCS using Equation (9). Two sets of MSS reported in Cox and Munk (C54) are also from sun glitter analysis but from a spaceborne optical sensor (wind speed up to 15 m/s); the results are essentially identical to those of the clean water condition in Cox and Munk [11]. For the slick waters, surface waves shorter than about 30 cm, corresponding to *k_u_* = 21 rad/m, are suppressed [11]; therefore, the two sets of optical MSS are $s_{\infty }^{2}$ and $s_{21}^{2}$. The Ku band (14 GHz) EM wavelength is about 2.1 cm, with the factor *k_r_*/*k_u_* = 3 to 6 applied, the observed LPMSS is between $s_{98}^{2}$ and $s_{49}^{2}$. The surface roughness sensed by the Ku band altimeter is expected to be between the optical data in clean and slick waters. This, however, is not the case for the result in low and moderate winds (*U*_10_ ≤ ~10 m/s), as illustrated in Figure 2b. Especially intriguing is that the discrepancy increases toward lower wind condition for which the ocean surface is less nonlinear and simplifications made in the EM theoretical development are better justified.

As mentioned in the Introduction, many researchers resort to using an effective reflectivity much smaller than that computed from the relative permittivity [8,9,10]; more discussion on reflectivity is deferred to Section 4.2. In Hwang et al. [13,14], the authors stress that Equation (8) carries the physical meaning of an exponentially attenuating contribution with respect to the incidence angle *θ_i_*. Borrowing the two-scale concept of scattering at moderate incidence angles [8] that the local incidence angle can be modified by the background waves, and reexamining Equation (6), the left-hand side of Equation (6) can be written as $\sigma_{0\xi \eta }^{}\left(\theta_{s},\gamma \right)$, and Equation (8) is explicitly written as

(11) $$\sigma_{0\xi \eta }\left(\theta_{s}=\theta_{i},\gamma \right)={\left|R_{\xi \eta }\left(\iota \right)\right|}^{2}\frac{\sec^{4}\gamma }{s_{f}^{2}}\exp\left(\frac{-\tan^{2}\gamma }{s_{f}^{2}}\right)$$

**Figure 3:**
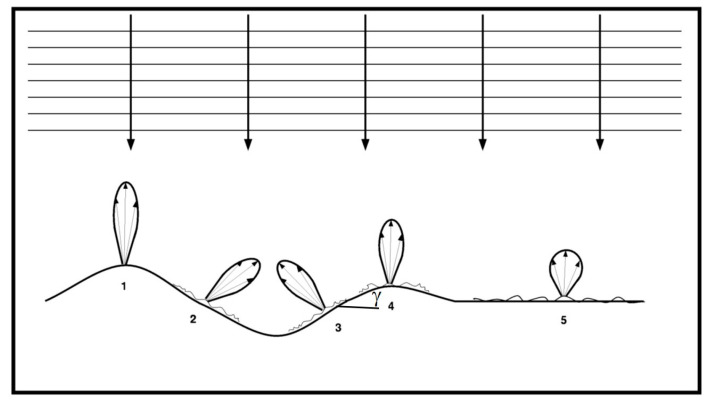
A conceptual sketch depicting the scattering of radar waves by surface patches of various roughness (1, 4, and 5) and the effect of tilting background surface on the backscattering intensity from patches of identical statistical roughness (2, 3, and 4); reproducing (Figure 7, [13]).

The alternative explanation offered in [13,14] is that Equation (8) can be interpreted as the specular scattering pattern with respect to the incidence angle *θ_i_*. This is illustrated in a conceptual sketch (Figure 7, [13]), which is reproduced as Figure 3 here. In the Figure 7 of Ref [13], the parallel horizontal lines represent the far-field EM wave fronts emitted from zenith and impinge on the ocean surface. Five scattering patterns are illustrated. Patterns 1, 4, and 5 are from three incrementally rougher patches located on background surfaces that are locally parallel to the incoming wave fronts such that the local incidence angle is not changed from the nominal incidence angle (0 in this case). The backscattering returns from the three patches are inversely proportional to the surface roughness as expected from Equation (9). Patterns 2, 3, and 4 are from three statistically identical roughness patches and located on background surfaces with different orientations. The backscattering strengths from the three patches observed by the antenna at zenith are different, although the reflecting patches have identical statistical roughness. The difference of the returns toward the nominal incidence direction (from zenith) reflects the tilting effect as described by the exponential term in Equation (8) or Equation (11).

Consequently, there is one more step to obtaining the backscattering NRCS accounting for modification of local incidence angle, that is,

(12) $$\begin{array}{ll} \sigma_{0\xi \eta }\left(\theta_{s}=\theta_{i}\right)= & \int_{-\infty }^{\infty }\int_{-\infty }^{\infty }{\left|R_{\xi \eta }\left(\iota \right)\right|}^{2}\frac{\sec^{4}\theta_{il}}{s_{f}^{2}}\exp\left(\frac{-\tan^{2}\theta_{il}}{s_{f}^{2}}\right).\\ & p\left(\tan\gamma_{x},\tan\gamma_{y}\right)d\tan\gamma_{x}d\tan\gamma_{y} \end{array}$$

where *θ_il_* is the local incidence angle satisfying the specular reflection condition (i.e., *θ_il_* = *θ_sl_* on the tilting surface, *θ_sl_* is the local scattering angle), *γ* is the global tilting angle, tan*γ* = (tan*γ_x_*, tan*γ_y_*) = (*ζ_x_*, *ζ_y_*) is the corresponding surface slope, and *p*(tan*γ_x_*, tan*γ_y_*) = *p*(*ζ_x_*, *ζ_y_*) is the probability density function (pdf) of the background (global) surfaces that tilt the specular patches. The term inside the double integrals and before the pdf function, that is essentially Equation (8), is now interpreted as the scattering pattern, and Equation (12) is equivalent to the tilting modulation of local incidence angle in the discussion of scatterometer returns [8] with the pdf function accounting for the off-specular contribution. For the Gaussian distribution of sea surface slopes with equal up-downwind and crosswind slope components, i.e., $s_{fx}^{2}=s_{fy}^{2}=s_{f}^{2}/2$,

(13) $$p\left(\tan\gamma_{x},\tan\gamma_{y}\right)=\;\frac{1}{\pi s_{f}^{2}}\exp\left(\frac{-\tan^{2}\gamma }{s_{f}^{2}}\right)$$

For the altimeter application, we have

(14) $$\begin{array}{ll} \sigma_{0}\left(0\right)= & \int_{-\infty }^{\infty }\int_{-\infty }^{\infty }{\left|R\left(0\right)\right|}^{2}\frac{\sec^{4}\gamma }{s_{f}^{2}}\exp\left(\frac{-\tan^{2}\gamma }{s_{f}^{2}}\right)\cdot \\ & \frac{1}{\pi s_{f}^{2}}\exp\left(\frac{-\tan^{2}\gamma }{s_{f}^{2}}\right)d\tan\gamma_{x}d\tan\gamma_{y} \end{array}$$

**Figure 4:**
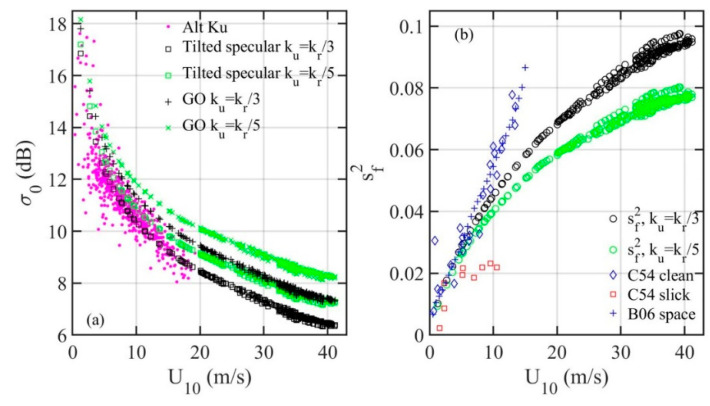
(**a**) Ku band NRCS and comparison with model computation with Equation (14), for comparison; computation results with Equation (9) are also shown (labeled GO). (**b**) The LPMSS used for the NRCS computation shown in (**a**), and comparison with the optical MSS in clean and slick waters. The LPMSS is computed with the H18 spectrum model integrated to *k_r_*/3 and *k_r_*/5.

Figure 4a shows the NRCS results computed with Equation (14) and their comparison with altimeter observations. For reference, the results computed with Equation (9) are also shown. Figure 4b shows the two sets of $s_{f}^{2}$ used in the computation. They are based on the H18 spectrum model ($s_{H18}^{2}$) [17,27] integrated to *k_u_* = *k_r_*/3 and *k_r_*/5. The agreement between measurement and theoretical computation using Equation (14) with H18 MSS integrated to *k_u_* = *k_r_*/3 is improved considerably. More discussion on LPMSS analysis and reflectivity |*R*(0)|^2^ is deferred to Section 4.

For specular returns, the wind direction enters into the mean square slopes in the up-downwind and crosswind components. The ratio of the crosswind to up-downwind components is close to one based on Cox and Munk’s optical sun glitter data [11], and our analysis assumes equal up-downwind and crosswind slope components. More discussion on the up-downwind and crosswind surface roughness components is also given in Section 4 of [17], see their Figure 10 and related discussion.

### 3.2. L Band CYGNSS Analysis

Tilting modification is expected to impact specular reflections at oblique angles. Applying the same procedure discussed in the altimeter case to Equation (6) for $\sigma_{0\xi \eta }^{}\left(\theta_{s},\gamma \right)$, the NRCS for 2D Gaussian pdf of tilting surfaces is:

(15) $$\begin{array}{ll} \sigma_{0}\left(\theta_{s}\right)= & \int_{-\infty }^{\infty }\int_{-\infty }^{\infty }{\left|R_{\xi \eta }\left(\iota \right)\right|}^{2}\frac{\sec^{4}\gamma }{s_{f}^{2}}\exp\left(\frac{-\tan^{2}\gamma }{s_{f}^{2}}\right)\cdot \\ & \frac{1}{\pi s_{f}^{2}}\exp\left(\frac{-\tan^{2}\gamma }{s_{f}^{2}}\right)d\tan\gamma_{x}d\tan\gamma_{y} \end{array}$$

It turns out that for global positioning system reflectometry (GPSR) with right-hand-circular transmit and left-hand-circular receive, the |*R_lr_*(*ι*)|^2^ is almost independent on local incidence angle, *ι*, up to about 50° [27], so |*R_lr_*(*ι*)|^2^ plays a rather minor role in computing circular polarization NRCS except at low grazing angles. The reflectivity in Equation (6) or Equation (15) can be approximated by |*R_lr_*(0)|^2^ for GPSR from the ocean surface.

**Figure 5:**
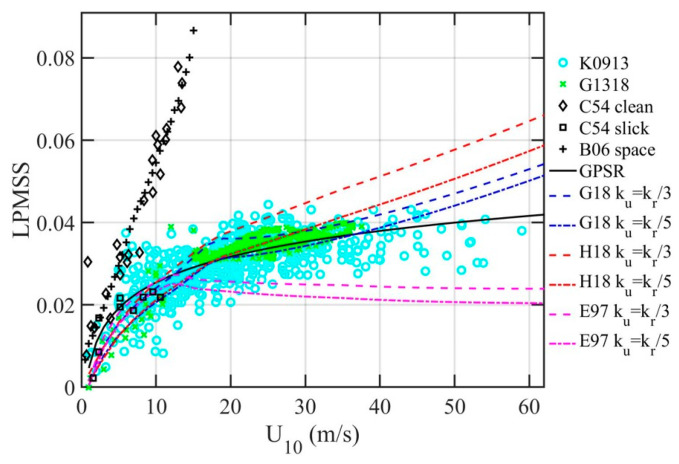
L band LPMSS derived from GPSR delay Doppler analyses (labeled K0913 and G1318), the least-squares curve fitted through all the GPSR data is given by the solid black line (labeled GPSR). Three sets of computation from spectrum models (E97, G18, and H18) with *k_u_* = *k_r_*/3 and *k_r_*/5 are also illustrated. For comparison, optical MSS in clean and slick waters is shown with black markers.

**Figure 6:**
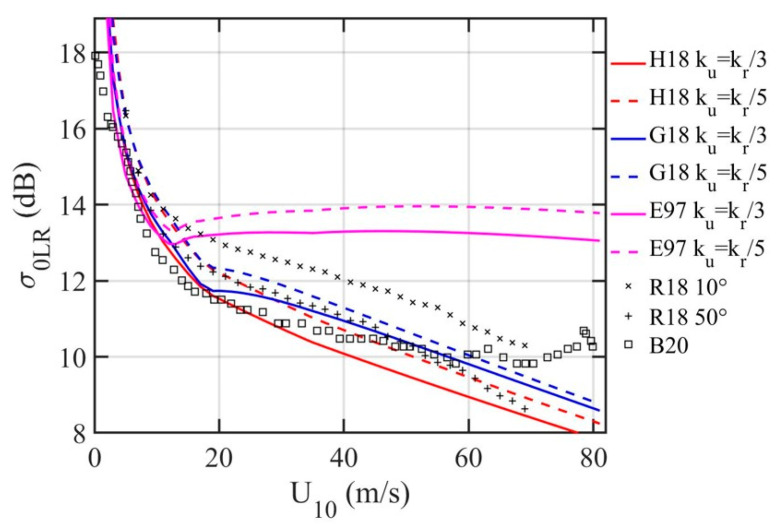
NRCS computed with LPMSSs derived from E97, G18, and H18 spectrum models, and comparison with the CYGNSS NRCS observations: R18 and B20.

Through GPSR delay Doppler waveform analyses, there are now several sets of L band LPMSS collected in TC conditions [18,19,20,21]. These data are identified as $s_{GPSR}^{2}$ and illustrated in Figure 5 (labeled K0913 and G1318 in the legend); the least-squares fitted curve is given by the solid black line (labeled GPSR). The $s_{GPSR}^{2}$ data have served to address one of the most unsettled issues in the ocean surface wind wave spectrum function *S*(*f*), i.e., the spectral slope in the high frequency region [17,28]. The clarification of spectral slope is especially critical to the LPMSS determination using an ocean wave spectrum model. The refinement of the *S*(*f*) function, in turn, offers the feasibility to derive LPMSS given *U*_10_ and windsea dominant wave period *T_p_* from operational-system measurements [29] or to create synthetic high-wind LPMSS data set with a small number of critical tropical cyclone (TC) parameters [17,27]. Also shown in Figure 6 are the LPMSS computed from the G18 ($s_{G18}^{2}$) and H18 ($s_{H18}^{2}$) spectrum models [17,27] with *k_u_* = *k_r_*/3 and *k_r_*/5. In addition, the results based on E97 ($s_{E97}^{2}$) [30] are also displayed; the E97 is used in the CYGNSS project [31,32]. For comparison, the optical data obtained in clean and slick sea surfaces [11,12] are illustrated with black markers in the Figure. The GPSR data have expanded the wind speed coverage considerably (from 15 to 59 m/s), and they are critical for refining the ocean surface wind wave spectrum models in high wind conditions. More detail on deriving LPMSS from a wave spectrum is deferred to Section 4.1.

Retrieving NRCS from GPSR, or global navigation satellite system reflectometry (GNSSR) in general, remains a challenging task [22,23,24]. Here we investigate the properties of L-band NRCS through forward computation using Equation (15). The results are then compared with the most recent publications of L band NRCS results from the CYGNSS mission [23,24]. Figure 6 shows the results computed with Equation (15) and the LPMSS derived from the three spectrum models shown in Figure 5. Superimposed with the computation curves are two sets of CYGNSS data: the black crosses and pluses labeled R18 [23] are the GMF at 10° and 50°, and the black squares labeled B20 [24] are the incidence-angle-averaged result. It is clear that the CYGNSS GMF is still evolving. Interestingly, there is a good agreement between the most recent version [24] (B20: black squares) and the computed NRCS with $s_{G18}^{2}$ and $s_{H18}^{2}$ integrated to *k_u_* = *k_r_*/3; the model-data difference is less than about 1 dB to wind speed up to about 60 m/s. The computed NRCS with $s_{E97}^{2}$ is also in good agreement with data for low to moderately high winds (*U*_10_ ≤ ~14 m/s) but fares far worse in higher winds, because E97 underestimates the LPMSS for *U*_10_ greater than about 14 m/s (Figure 5). It illustrates the importance of choosing an accurate surface wave spectrum model for ocean remote sensing analysis. More discussion on LPMSS and reflectivity |*R_ζη_*(*ι*)|^2^ is deferred to Section 4.

## 4. Discussion

### 4.1. LPMSS, Wind Speed, and Wave Development Stage

The functional form of the ocean surface wind wave spectrum remains one of the most uncertain quantities in ocean remote sensing problems. For specular return, the relevant property is the LPMSS with the upper integration wavenumber *k_u_* determined by the EM wavenumber *k_r_*. From altimeter analyses [9,10] the range of *k_r_*/*k_u_* is generally determined to be between 3 and 6. In this paper, we have shown results of specular computation obtained with *k_r_*/*k_u_* = 3 and 5, which corresponds to *k_u_* = 11 and 6.6 rad/m for L band (1.575 GHz), and 98 and 59 rad/m for Ku band (14 GHz). The contribution of long surface waves in the energetic dominant wave region becomes more important as wind speed increases and EM frequency decreases.

The value of the wave spectral slope *–s* in the high frequency region of the wind wave frequency spectral function is one of the most uncertain spectral properties critical to the determination of LPMSS. Traditional wind wave spectrum models assume *s* to be either 4 or 5 [33,34,35,36], although field observations have shown a wide range between about 2 and 7 [17,28]. The wave height spectral level drops sharply toward both high and low frequencies from the spectral peak; therefore, wave height measurements are not very sensitive for addressing the spectral slope issue. Wave slope data are much more useful for this task [27,29].

For many decades, the airborne sun glitter analyses in clean and slick waters reported in 1954 [11] have remained the most comprehensive ocean surface MSS dataset; the wind speed range is between 0.7 and 13.5 m/s for the clean water condition, and between 1.6 and 10.6 m/s for the slick water condition. The spaceborne sun glitter analysis reported in 2006 [12] expands slightly the wind speed range of clean water condition to about 15 m/s, and the results are essentially identical to those of the clean water condition reported in 1954 [11]. The recent results of $s_{GPSR}^{2}$ further extend the LPMSS wind speed coverage to 59 m/s [18,19,20,21]. With the EM frequency of 1.575 GHz, the *k_u_* is between about 5 and 11 rad/m, and $s_{GPSR}^{2}$ data are most useful for investigating the wind wave spectrum slope. The study leads to establishing a general wind wave spectrum function G18 [17], with the applicable upper limit (*k_max_*) of the G18 spectrum function estimated to be about the upper range of L band *k_u_* (11 rad/m). For Ku band application, the hybrid model H18 is more suitable. The H18 model uses G18 for long waves and H15 [16] for short waves, with linear interpolation between *k* = 1 and 4 rad/m [27].

**Figure 7:**
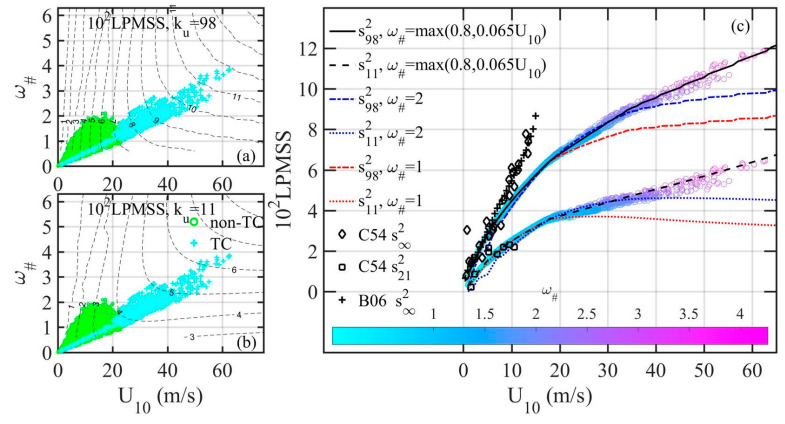
Pre-calculated H18 LPMSS contour maps for (**a**) *k_u_* = 98 rad/m and (**b**) *k_u_* = 11 rad/m. Superimposed in the Figures are the observed *ω_#_*(*U*_10_) in TC and non-TC conditions. (**c**) Color markers show the interpolated LPMSS for *k_u_* = 98 and 11 rad/m (upper and lower sets, respectively) using the observed *ω_#_*(*U*_10_) in TC and non-TC conditions. For comparison, illustrated with black markers are the optically sensed MSS in clean and slick waters. Also shown in the Figure are the interpolated LPMSS assuming constant *ω_#_* (1 and 2), and *ω_#_* approximated by Equation (16).

Wind speed *U*_10_ and windsea dominant wave period *T_p_* are the only required input for computing the G18 and H18 spectrum (and many other spectrum models such as the E97 discussed in Section 3). The combination of *U*_10_ and *T_p_* can be expressed as the dimensionless spectral peak frequency *ω_#_* = *U*_10_/*c_p_* = *U*_10_/(*gT_p_*/2π), where *c_p_* is the wave phase speed of the spectral peak component, and *g* is gravitational acceleration. The inverse of *ω_#_* is wave age, which represents the stage of wave development. Determining the wave spectrum requires consideration of both wind speed and wave development stage. With a wave spectrum function, the $s_{ku}^{2}$ can be pre-calculated for a range of *U*_10_, *ω_#_*, and *k_u_*. For example, Figure 7a,b show the contour maps of H18 $s_{98}^{2}$ and $s_{11}^{2}$, respectively. They are illustrated for *U*_10_ between 0 and 70 m/s, and *ω_#_* between 0.8 and 5.2. These pre-calculated results serve as design curves or lookup tables for quickly obtaining the desired $s_{f}^{2}$ through interpolation. Superimposed in the Figures are the observed *ω_#_*(*U*_10_) in TC and non-TC conditions. Because the wave age 1/*ω_#_* is defined as the ratio of dominant wave phase speed *c_p_* and wind speed *U*_10_, *U*_10_, and *ω_#_* = *U*_10_/*c_p_* are not independent variables. The observed *ω_#_*(*U*_10_) in TC and non-TC conditions show the general linear relationship with a narrow range of *ω_#_* variation for a particular wind speed *U*_10_ as illustrated by the color markers in Figure 7a,b.

**Figure 8:**
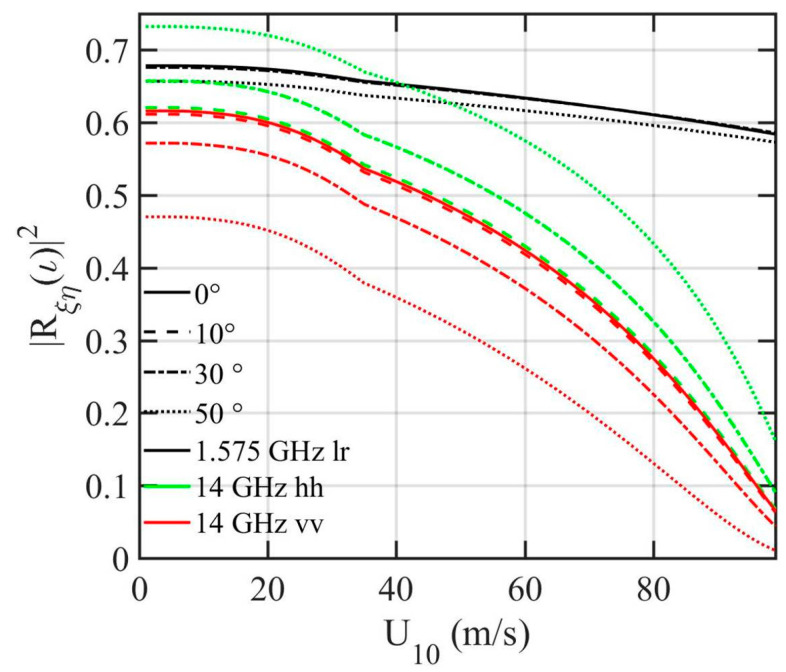
The sea surface reflectivity at 0°, 10°, 30°, and 50° incidence angles (solid, dashed, dashed–dotted, and dotted curves, respectively) for Ku (14 GHz, green and red curves) and L (1.575 GHz, black curves) bands. The GPSR signals are right-hand-circular transmit and left-hand-circular receive, so for L band |*R_lr_*(*ι*)|^2^ is given (black curves). For Ku band, the reflectivity for *hh* and *vv* polarizations is shown (green and red, respectively).

The interpolated H18 $s_{98}^{2}$ and $s_{11}^{2}$ are presented with color markers in Figure 8. For comparison, the optically sensed $s_{\infty }^{2}$ in clean water [11,12] and $s_{21}^{2}$ in slick water [11] are illustrated with black markers in the Figure. Also shown in Figure 8 are the interpolated H18 $s_{98}^{2}$ and $s_{11}^{2}$ assuming constant *ω_#_* (1 and 2 are used for illustration). As wind speed increases, the difference increases between $s_{f}^{2}$ computed with constant and observed *ω_#_*. Interestingly, if the approximation

(16) $$\omega_{\#}=\max\left(0.8,0.065U_{10}\right)$$

is employed, the resulting $s_{f}^{2}$ is very close to that obtained with observed *ω_#_*. For the purpose of obtaining $s_{f}^{2}$ from a wave spectrum function, approximation Equation (16) simplifies the procedure in practical applications, since it requires only the *U*_10_ input (with *k_u_* specified).

### 4.2. Surface Reflectivity Considering Wave Breaking

The reflectivity |*R_ξη_*(*ι*)|^2^ is a function of relative permittivity and generally treated as a constant for a given EM frequency (with the assumption of some representative sea surface temperature and sea surface salinity: 293 K and 35 psu are used throughout this paper; the relative permittivity of sea water is computed with the formula given by Klein and Swift [37]). In high winds when air is entrained by wave breaking into the water surface layer and foam covers the water surface, the modification of relative permittivity by the mixed air needs to be considered. Figure 8 shows the Fresnel reflectivity at 0°, 10°, 30°, and 50° incidence angles for Ku (14 GHz) and L (1.575 GHz) frequencies. The Ku band altimeters discussed in this paper operate with linear polarizations (*h* and *v*), so |*R_hh_*(*θ*)|^2^ and |*R_vv_*(*θ*)|^2^ are illustrated. The GPSR signals are right-hand-circular transmit and left-hand-circular receive, so for L band |*R_lr_*(*ι*)|^2^ is given; its dependence on incidence angle is very weak up to about 50° incidence angle [27]. For altimeter reflection from the ocean surface, |*R_ξη_*(0)|^2^ is the quantity of interest (Section 3.2), and it is independent of polarization states *hh*, *vv*, and *lr*, but can vary considerably with wind speed as a result of air entrainment by wave breaking. The foam modification is more severe toward higher frequency as expected; this can be seen from comparing the black solid line of L band and red/green (overlapped) solid lines of Ku band. The procedure to account for the foam effect is briefly described below.

Through analyses of microwave radiometer measurements collected in TCs that cover a wide range of frequencies, incidence angles, and both horizontal and vertical polarizations [38,39,40,41,42,43,44,45,46], the effects of surface foam generated by wave breaking are expressed as a function of wind speed, microwave frequency, and incidence angle [47,48]. The effective air fraction *F_a_* is related to the whitecap coverage *W_c_* as described in Appendices A and B of [48]. A brief summary is given here. The proposed *F_a_*/*W_c_* function is

(17) $$\frac{F_{a}}{W_{c}}=\max\left[1,{\left(\frac{f}{f_{ref}}\cos^{\alpha }\theta \right)}^{\beta }\right]$$

From empirical fitting the computed brightness temperature with observations [38,39,40,41,42,43,44,45,46], the following values are recommended for the three parameters in Equation (17):

*f_ref_* = 14 GHz,

*α* = 1.3, and

$$\beta =\max\left\{0,0.5-\min\left\{0.5,0.5\left[\exp\left(1.1f/f_{ref}\right)-1.5\right]\right\}\right\}$$

The whitecap fraction *W_c_*(*U*_10_) is defined by

(18) $$W_{c}=\left\{\begin{array}{cc} 0, & u_{*}\leq 0.11\;m/s\\ 0.30{\left(u_{*}-0.11\right)}^{3}, & 0.11<u_{*}\leq 0.40\;m/s\\ 0.07u_{*}^{2.5}, & u_{*}>0.40\;m/s \end{array}\right.$$

where $u_{*}$ is the wind friction velocity; the drag coefficient connecting $u_{*}$ and *U*_10_ input ($u_{*}=C_{10}^{0.5}U_{10}$) is given by

(19) $$C_{10}=\left\{\begin{array}{cc} 10^{-4}\left(-0.0160U_{10}^{2}+0.967U_{10}^{}+8.058\right), & U_{10}\leq 35m/s\\ 2.23\times 10^{-3}{\left(U_{10}/35\right)}^{-1}, & U_{10}>35m/s \end{array}\right.$$

With the effective air fraction *F_a_* determined from the whitecap coverage *W_c_*, the effective relative permittivity *ε_e_* is computed with the refractive mixing rule [49,50,51]:

(20) $$\varepsilon_{e}={\left[F_{a}\varepsilon_{a}^{1/2}+\left(1-F_{a}\right)\varepsilon_{sw}^{1/2}\right]}^{2}$$

where *ε_a_* and *ε_sw_* are the relative permittivities of air and (foamless) sea water, respectively. The effective relative permittivity *ε_e_* is then used to compute the Fresnel reflection coefficient with wind speed dependence as a result of foam effects caused by surface wave breaking.

### 4.3. Frequency Dependence and Verification

**Figure 9:**
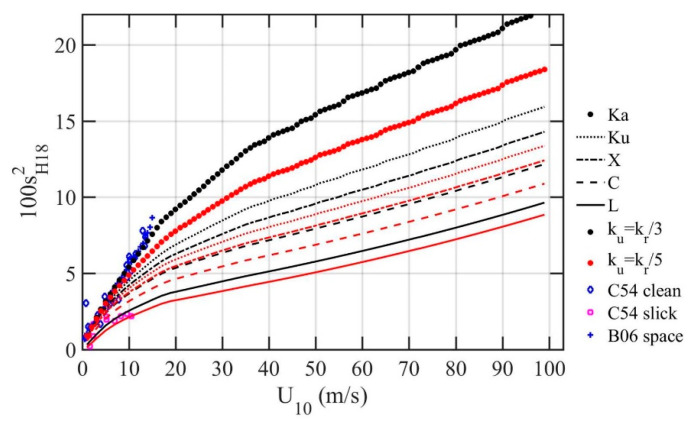
LPMSS for L (1.575 GHz, solid lines), C (6 GHz, dashed), X (10 GHz, dashed-dotted), Ku (13.575 GHz, dotted), and Ka (35.75 GHz, dots) bands based on the H18 spectrum (for normal incidence application), black curves are integration to *k_r_*/3 and red curves are integration to *k_r_*/5.

**Figure 10:**
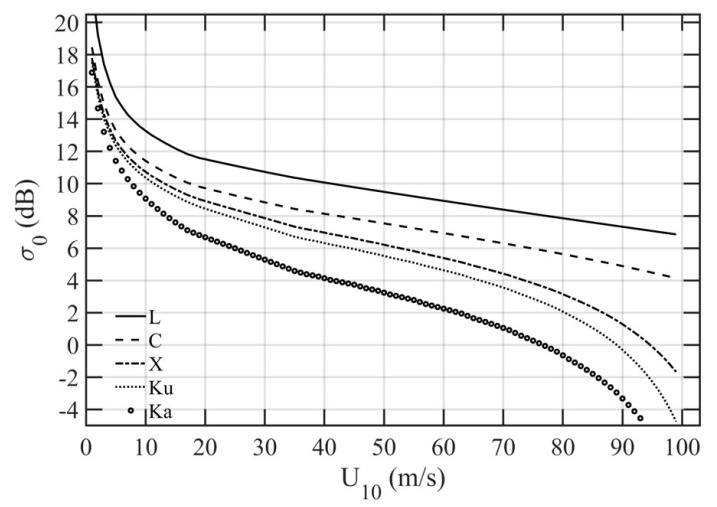
Specular NRCS (normal incidence) dependence on wind speed for L (1.575 GHz), C (6 GHz), X (10 GHz), Ku (14 GHz), and Ka (35.75 GHz) bands. All computed with the H18 LPMSS integrated to *k_r_*/3.

With a surface wave spectrum, the LPMSS can be computed for application to any EM frequencies. For example, Figure 9 shows $s_{H18}^{2}$ integrated to *k_u_* = *k_r_*/3 (black curves) and *k_u_* = *k_r_*/5 (red curves) for several microwave frequency bands used frequently in ocean remote sensing (L, C, X, Ku, and Ka) with wind speeds up to 99 m/s. Also illustrated for comparison is the optical MSS, which is restricted to wind speed below about 15 m/s. The specular NRCS can be computed with the LPMSS input, as illustrated in Figure 10. For clarity, only results based on $s_{H18}^{2}$ integrated to *k_u_* = *k_r_*/3 are shown. It is of interest to verify the model computations with available data.

The Ku band altimeter systems have many more well-calibrated NRCS datasets compared to other frequencies due to the long history of using Ku band for ocean wind sensing [9,10,13,14,52,53,54,55,56,57,58,59,60,61,62,63,64]. Three examples are examined here:

1. TOPEX/POSEIDON (T/P) altimeter NRCS (13.575 GHz) and collocated National Oceanographic and Atmospheric Administration (NOAA) National Data Buoy Center (NDBC) buoy datasets in three geographic regions (Gulf of Alaska and Bering Sea, Gulf of Mexico, and Hawaii islands) have been reported in [13,14] with up to seven years of simultaneous measurements and a total of 2174 (*U*_10_, *σ*_0_) pairs. The maximum wind speed in the datasets is about 20 m/s. The maximum temporal and spatial differences between buoy and altimeter data are 0.5 h and 100 km, respectively. These data are referred to as H02 from here on.
2. A one-year Tropical Rainfall Mapping Mission (TRMM) Precipitation Radar (TPR, 13.8 GHz) dataset [10] with more than 1.13 × 10^7^ (*U*_10_, *σ*_0_) pairs. The TPR dataset is quite unusual because the wind sensor and altimeter are on the same satellite. The nadir footprint of the TRMM Microwave Imager (TMI) wind sensor is collocated with the footprint of the TPR, and there is no need for temporal interpolation. The spatial resolution of TPR altimeter is about 4.3 km and that of the TMI is about 25 km, so the spatial separation between TPR, NRCS, and TMI wind speed data is no more than ± 12.5 km. The maximum wind speed in the dataset as presented in their Figure 4 is about 29 m/s. These data are referred to as F03 from here on.
3. An extensive collection of 33 years wind speed, wave height, and altimeter NRCS from 13 satellite missions ranging from Geosat to Sentinel-3A and Jason-3 [64]. The altimeter NRCS is merged with European Centre for Medium-Range Weather Forecasts (ECMWF) model wind speed in 1° × 1° grids; their Figure 3 shows an example of the Jason-3 (J3) NRCS dependence on wind speed. The maximum wind speed is about 20 m/s. These data are referred to as R19 from here on.

**Figure 11:**
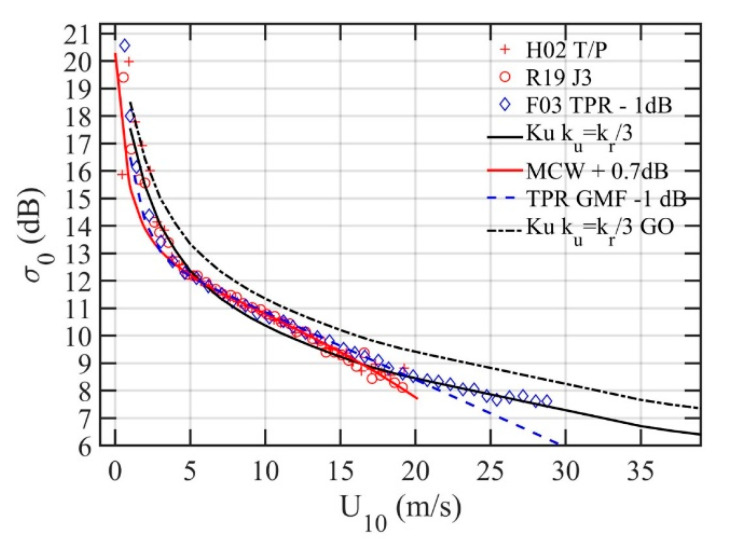
Ku band altimeter NRCS from T/P, J3, and TPR missions (normal incidence) and comparison with NRCS computations using the H18 LPMSS integrated to *k_r_*/3 (black solid line). The MCW and TPR GMFs (with adjustment) are shown with red solid and blue dashed lines; more detail is given in the text.

Figure 11 shows the bin-averaged Ku band altimeter data from the three examples described above, the data are labeled H02, F03, and R19. The H02 T/P results (red pluses) are processed from our in-house data sets. The R19 J3 results (red circles) are digitized from their Figure 3 [64]. The F03 TPR results (blue diamonds) are digitized from their Figure 4 [10]. Compared to the T/P and J3 results, there is a 1-dB systematic bias in the TPR data, which is subtracted in the Figure. A 1.92 dB bias is reported [10] from comparing with the modified Chelton and Wentz (MCW) GMF (Table 1, [59]). The MCW is designed for the Geosat altimeter. With improved algorithm and including atmospheric correction, the T/P NRCS differs from the Geosat NRCS by 0.7 dB [54]. The MCW GMF with 0.7-dB adjustment is shown with the red solid line, which goes through the center of T/P, J3, and adjusted TPR data. The blue dashed line is the TPR GMF with 1 dB adjustment applied. The black line is the model computation with $s_{H18}^{2}$ integrated to *k_u_* = *k_r_*/3. The agreement is very good between model computation and all three Ku band data sets. In high winds, the model computation even outperforms the empirically established GMFs. Also shown for reference is the GO solution Equation (9) with the black dashed line, which is about 1 dB too high compared to the data.

**Figure 12:**
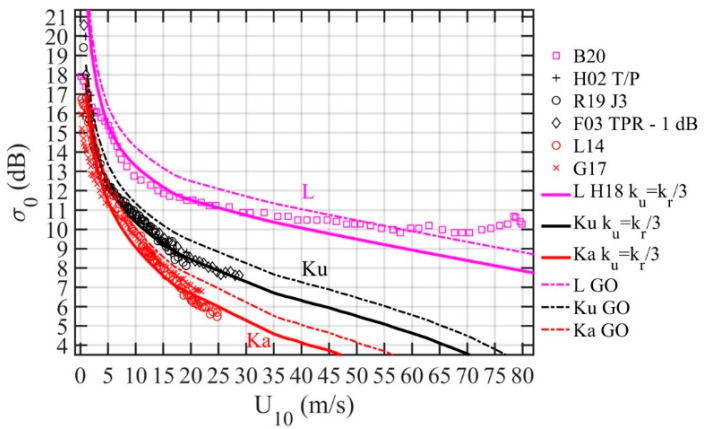
Model–measurement comparison for L, Ku, and Ka microwave frequencies; more detail is given in the text.

Figure 12 shows the data from all three EM frequencies together and the comparison with model computation using (14) with $s_{H18}^{2}$ integrated to *k_u_* = *k_r_*/3. The number of reported data sets is considerably less in other EM frequencies. As mentioned in Section 3, there are two L band NRCS data sets reported from the CYGNSS mission [23,24]. A couple of Ka band altimeter (ALtiKa) data sets have also been reported from the SARAL mission [25,26]. For the Ka band, the model-data agreement (red markers and solid line) is close to that of the Ku band comparison (black markers and solid line). For L band, the model results with $s_{H18}^{2}$ integrated to *k_u_* = *k_r_*/3 are shown (magenta marker and solid line). Less than 1 dB difference is found between model and measurements to wind speed about 60 m/s. Also shown for reference are the GO solutions Equation (9) for the three frequencies illustrated with dashed lines, which are about 1 dB too high compared to the data.

### 4.4. Incidence and Scattering Angle Dependence

**Figure 13:**
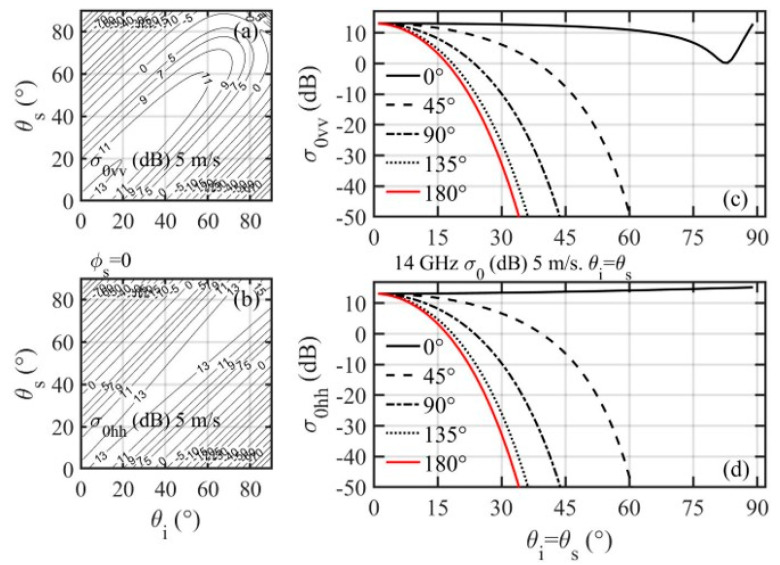
Examples of NRCS dependence on incidence and scattering angles. In-plane forward scattering (*ϕ_s_ =* 0°): (**a**) *vv*, (**b**) *hh*. Variation of NRCS along *θ_i_ = θ_s_* for *ϕ_s_ =* 0°, 45°, 90°, 135°, and 180° scattering azimuth angles: (**c**) *vv*, (**d**) *hh*. The microwave frequency is 14 G Hz and wind speed is 5 m/s.

**Figure 14:**
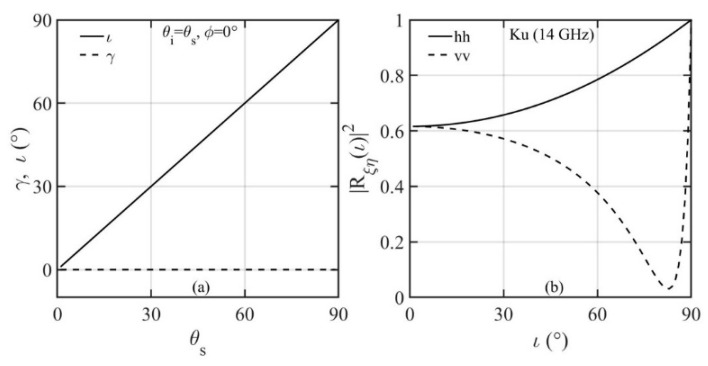
Relevant parameters governing in-plane specular reflection (*θ_i_ =θ_s_*, *ϕ_s_ =* 0°): (**a**) local incidence angle *ι* and facet angle *γ*, (**b**) surface reflectivity.

The NRCS dependence on the incidence and scattering angles hinges on *ι* and *γ* as prescribed by Equation (3) and Equation (7). Figure 13a,b show examples of in-plane (*ϕ_s_* = 0°) *vv* and *hh* scattering of Ku band (EM frequency *f* is 14 GHz) at 5 m/s wind speed. For clarity, the contour lines are 2 dB apart for NRCS greater than 5 dB, 5 dB apart between −30 and 5 dB, and 10 dB apart for NRCS less than −30 dB. The NRCS contours are symmetric to the *θ_i_* = *θ_s_* line. This is a consequence of reciprocity, since we get the same set of (*ι*, *γ*) from Equation (3) and Equation (7) when *θ_i_* and *θ_s_* are swapped. The dropoff along the *θ_i_* = *θ_s_* line is relatively mild: about 2.1 dB between *θ_i_* = *θ_s_* = 0° and *θ_i_* = *θ_s_* = 60°. The NRCS dropoff with respect to Δ*θ* = *θ_i_* − *θ_s_* is dependent on incidence angle. For example, with *θ_i_* = 0°, the NRCS difference is about 4 dB for *θ_s_* from 0° to 20°, and much steeper at large *θ_s_*, about 14 dB for the same Δ*θ* with *θ_s_* from 20° to 40°. Figure 13c,d show variation of *vv* and *hh* NRCS along *θ_i_ = θ_s_* for scattering azimuth angles *ϕ_s_ =* 0°, 45°, 90°, 135°, and 180°. Interestingly, in the in-plane condition (*ϕ_s_ =* 0°, solid black lines in Figure 13c,d), the SPT predicts strong NRCS even at low grazing angles. For this configuration (*θ_i_ = θ_s_*, *ϕ_s_ =* 0°), *γ =* 0°, *ι = θ_i_ = θ_s_* (Figure 14a), the in-plane specular forward scattering is just like altimeter that reflects from flat facets (*γ =* 0°), except that now the effective incidence angle is *ι = θ_i_ = θ_s_*, and the NRCS is $\sigma_{0\xi \eta }={\left|R_{\xi \eta }\left(\iota \right)\right|}^{2}/s_{f}^{2}$. For the same ocean surface roughness, it varies only with the surface reflectivity (Figure 14b). Bistatic radars have been used in establishing forward-scatter fences for air and sea craft detection based on the strong forward scattering principle [65,66,67,68,69]. In the out-of-plane conditions (*ϕ_s_ ≠* 0°), the dropoff toward grazing is rather rapid at large *θ_i_ = θ_s_*. More discussion on the dependence on scattering azimuth angle is given later.

**Figure 15:**
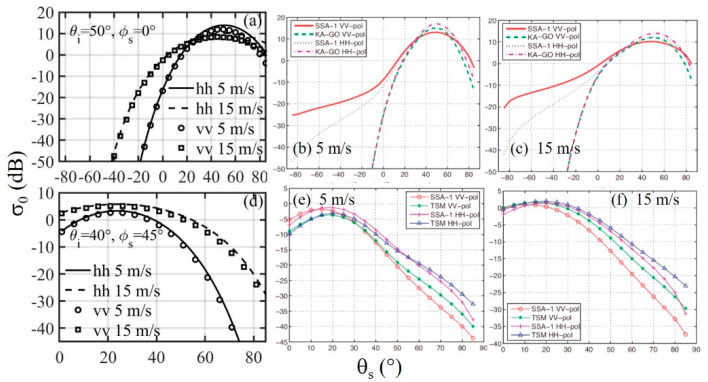
(Top row) Examples of in-plane NRCS dependence on *θ_s_*, *f* = 14 GHz, *θ_i_* = 50°. (**a**) Specular point computation, *U*_10_ = 5 and 15 m/s, (**b**) 5 m/s, and (**c**) 15 m/s SSA and GO solutions reproduced from Figure 3 of A06. (Bottom row) Examples of out-of-plane forward scattering (*ϕ_s_ =* 45°) NRCS dependence on *θ_s_*, *f* = 14 GHz, *θ_i_* = 40°. (**d**) Specular point computation, *U*_10_ = 5 and 15 m/s, (**e**) 5 m/s, and (**f**) 15 m/s SSA and GO solutions reproduced from Figure 6a and Figure 7a, respectively, of A06.

Figure 15a shows the in-plane (*ϕ_s_* = 0°) NRCS dependence on *θ_s_* for *θ_i_* = 50°, *U*_10_ = 5, and 15 m/s. In this figure, positive *θ_s_* is in the forward scattering direction and negative *θ_s_* is in the backward scattering direction. As expected, the maximum NRCS is at *θ_s_* = 50°, and the magnitude is larger for the lower wind condition with smoother surface roughness. The scattering beamwidth is narrower for the smoother surface roughness, so when *θ_s_* is different from *θ_i_* and with large |Δ*θ*|, the NRCS is larger in higher wind than that in lower wind. The *hh* NRCS is larger than the *vv* NRCS as a result of the larger *hh* reflectivity compare to *vv* (Figure 8). The SPT is basically following a geometric optics approach. A comparison of GO, small slope approximation (SSA), and two scale model (TSM) solutions is given in [70], here referred to as A06. Figure 3 of A06 is reproduced here as Figure 15b,c for 5 and 15 m/s, respectively. They give the results of SSA and GO comparison for the EM and wind conditions used in Figure 15a; the E97 spectrum [30] is used in the A06 analysis. Figure 15c can be compared with Figure 15a 5 m/s results given with solid line (*hh*) and circle marker (*vv*), and Figure 15d can be compared with Figure 15a 15 m/s results given with dashed line (*hh*) and square marker (*vv*). There is a good quantitative and qualitative agreement between the SPT and GO results.

Figure 15d shows the out-of-plane NRCS dependence on *θ_s_* for *θ_i_* = 40° and *ϕ_s_* = 45°. The high wind NRCS is larger than that of the low wind because of the broader beamwidth of scattering from the rougher surface. The SSA and TSM solutions for the conditions given in Figure 15d are given in Figure 6a and Figure 7a of A06, which are reproduced as Figure 15e,f, respectively, for 5 and 15 m/s. The agreement between SPT and TSM or SSA is fair. The difference between SPT and TSM or SSA is comparable to the difference between TSM and SSA. All three produce similar results near the specular angle.

**Figure 16:**
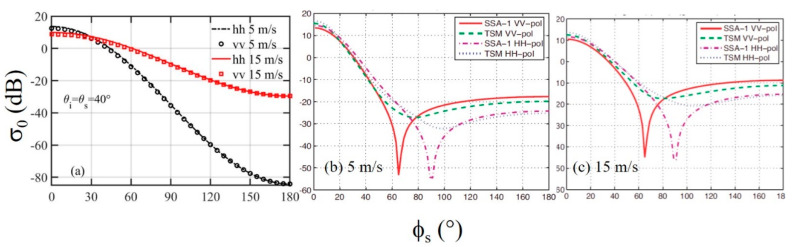
(**a**) Examples of NRCS dependence on *ϕ_s_*, *f* = 14 GHz, *θ_i_* = *θ_s_* = 40°, *U*_10_ = 5 and 15 m/s; (**b**) and (**c**) are the SSA and TSM solutions reproduced from Figure 8 of A06 for *U*_10_ = 5 and 15 m/s, respectively.

Figure 16a shows the NRCS dependence on the scattering azimuth angle *ϕ_s_*; the incidence and scattering elevation angles are fixed at *θ_i_* = *θ_s_* = 40°. The SPT gives a monotonically decreasing NRCS with respect to *ϕ_s_*, because the computed *ι* and *γ* vary with *ϕ_s_* monotonically. Figure 16b,c show the SSA and TSM solutions reproduced from Figure 8 of A06 for *U*_10_ = 5 and 15 m/s, respectively. There is a lobe structure especially pronounced in the SSA solution. The SPT is not able to produce such a lobe variation in the scattering azimuthal dependence.

The GO approach (SPT included) is applied to very rough surface accounts for the large-scale roughness [71]. For nadir-looking (altimeter), it works well for small incidence angles, up to about 15°. As incidence angle increases, the Bragg scattering from small-scale roughness becomes important, and methods such as TSM, SSA, and small perturbation method (SPM) have been developed [8,70,72,73,74]. These models are used for forward computation of the scattering coefficients of the sea surface as a function of various parameters, such as the angles of incidence for different frequencies, polarizations, wind speeds, and wind directions. Others are dedicated to the inversion problems where empirical methods are usually used to estimate the wind speed and sea state from altimeters, scatterometers (including SAR images), and more recently the bistatic reflectometers such as the GNSSR [23,24,75,76,77,78,79].

From the computed examples presented here, the SPT has been based on the geometric optics approach, has limited validity. Comparison with the GO, TSM, and SSA solutions suggests that the SPT works well for small |Δ*θ*| = |*θ_i_ − θ_s_*| and within a narrow azimuthal angle range in the forward specular scattering direction, estimated to be within about ±10° to ±20° in |Δ*θ*| and |*ϕ_s_*| (*ϕ_i_* = 0°). The applicable incidence angle range is unclear, but comparison with the SSA and TSM solutions [70] as shown in Figure 15 and Figure 16 suggests that the SPT gives reasonable results near the specular reflection direction for incidence elevation angle up to about 50° but maybe beyond, even to low grazing angle for the in-plane specular forward scattering condition (*θ_i_ = θ_s_*, *ϕ_s_ =* 0°). Despite its limitation, the computation of SPT is relatively simple, and it elicits a clear connection between EM scattering and the ocean surface properties.

## 5. Conclusions

The specular point theory [4,5,6,8] establishes a firm relationship between specular NRCS and surface wave statistical and geometric properties in a closed-form expression for a given *γ* (7). Specifically, it states that the NRCS is linearly proportional to the reflectivity |*R_ξη_*(*ι*)|^2^, inversely proportional to the LPMSS $s_{f}^{2}$, and multiplied with a term dominated by the exponential attenuation with respect to the surface slope at the specular point (tan*γ*). To consider the local incidence angle modification, the left-hand side of Equation (6) is expressed as $\sigma_{0\xi \eta }^{}\left(\theta_{s},\gamma \right)$. For small incidence and reflection angles, the modification of the local incidence angle can be significant as a result of the exponential term. To account for the local angle modification by the background tilting, a straightforward 2D Gaussian integration of Equation (6) is performed, which leads to Equation (14) for altimeters and Equation (15) for oblique reflectometers. The model results are in very good agreement with a broad collection of specular NRCS data from L, Ku, and Ka band instruments (Figure 11 and Figure 12).

We emphasize that remote sensing measurements are indisputably among the most important data sources of ocean surface roughness. The satellite platforms allow data collection in extreme wind conditions from global oceans. Such capability is unimaginable for traditional in-situ ocean sensors. Since ocean remote sensing is interdisciplinary, it requires coherent consideration from both remote sensing and oceanographic perspectives in order to access this precious ocean data source for improving the ocean surface wave spectrum that is important to remote sensing analysis. In return, the improved ocean surface wave spectrum provides more accurate forward computations of microwave altimeters, reflectometers, scatterometers, and radiometers covering a wide range of frequencies, incidence angles, and polarizations. The study presented here offers a method to use specular microwave returns for understanding the mean square slopes of ocean surface roughness several times longer than the radar wavelengths. There are plenty of spaceborne altimeter and reflectometer measurements in existence. These measurements, coupled with the SPT, represent a valuable data source of the ocean surface wave properties covering a wide range of wind conditions. There are limitations associated with the general geometric optics approach. The applicable incidence angle range based on our comparison with SSA and TSM solutions suggests that the SPT gives good results near the specular reflection direction, within about ±10° to ±20° in |Δ*θ*| = |*θ_i_* − *θ_s_*| and |*ϕ_s_*| (*ϕ_i_* = 0°), for incidence elevation angle up to about 50° but maybe beyond, even to low grazing angle for the in-plane specular forward scattering condition (*θ_i_ = θ_s_*, *ϕ_s_ =* 0°). The SPT provides a clear physical interpretation between the microwave scattering from a rough surface and the statistic and geometric properties of the rough surface. The computation of SPT is relatively simple and the results presented here suggest its usefulness for the interpretation of satellite altimeter and reflectometer data for the purpose of retrieving the surface wave properties, particularly the LPMSS.

## Data Availability

Data sets used in this analysis are given in the references cited. The processing codes and data segments can also be obtained by contacting the corresponding author.
